# Neuropsychological Investigation in Chinese Patients with Progressive Muscular Atrophy

**DOI:** 10.1371/journal.pone.0128883

**Published:** 2015-06-04

**Authors:** Bo Cui, Liying Cui, Mingsheng Liu, Xiaoguang Li, Junfang Ma, Jia Fang, Qingyun Ding

**Affiliations:** 1 Department of Neurology, Peking Union Medical College Hospital, Chinese Academy of Medical Sciences, Beijing, China; 2 Neurosciences Center, Chinese Academy of Medical Sciences, Beijing, China; Institute of Health Science, CHINA

## Abstract

**Background:**

Progressive muscular atrophy (PMA) is a rare type of degenerative motor neuron disease (MND) of which the onset happens in adult period. Despite its well-defined clinical characteristics, its neuropsychological profile has remained poorly understood, considering the consensus of cognitive and behavioral impairment reached in amyotrophic lateral sclerosis (ALS).

**Methods:**

We conducted a cross-sectional evaluation of Chinese PMA patients with a series of comprehensive batteries emphasizing the executive and attention function, and covering other domains of memory, language, visuospatial function, calculation and behavior as well. Their performances were compared with those of age- and education-matched ALS and healthy controls (HC).

**Results:**

21 patients newly diagnosed with PMA were consecutively enrolled into our ALS and other MND registry platform, accounting for 14.7% of all the incident MND cases registered during the same period. 20 patients who completed the neuropsychological batteries were included into analysis. Compared with HC, PMA performed significantly worse in maintenance function of attention, while they exhibited quantitative similarity to ALS in all behavioral inventories and neuropsychological tests except the time for Stroop interference effect.

**Conclusion:**

PMA could display mild cognitive dysfunction in the same frontal-mediated territory of ALS but in a lesser degree, whereas they did not differ from ALS behaviorally.

## Introduction

Progressive muscular atrophy (PMA) is an adult onset progressive neurological disease, clinically manifested by prominent lower motor neuron (LMN) involvement and the absence of upper motor neuron (UMN) features, yet pathologically appearing as a variant of amyotrophic lateral sclerosis (ALS) [1]. It has been shown that ALS is subject to a neurodegenerative spectrum beyond motor neurons: sharing similar genetic and molecular basis with frontotemporal lobe degeneration (FTLD), up to 50% of patients with ALS have demonstrated cognitive and/or behavioral impairment, especially in frontal-mediated territory, and 10% to 15% of the patients could display frank dementia [2–4]. Thus it is a reasonable hypothesis that patients with PMA could exhibit the same pattern of deficit with that of ALS if they belong to the same pathological category. However, most relevant researches for ALS adopted 1998 revised El Escorial criteria [5] which excluded the category of “suspected ALS”[6], and consequently PMA was not included into analysis. Bias might arise for the aim of depicting the neuropsychological profile in the context of motor neuron disease (MND), considering PMA accounting for around 10% of MND [7] and their potential of turning into ALS with UMN signs appearing [8]. Besides, clarifying this issue would in turn assist to confirm whether ALS and PMA are heterogeneous or not.

These facts contributed to the need of expanding investigations to the relative less frequent subtype of MND. Hence, our object is to characterize cognitive and behavioral status in PMA, and to testify whether PMA and ALS belong to a same entity by comparing them with ALS and healthy controls (HC). By this effort, we hope to provide more comprehensive description of Chinese patients with MND.

## Methods

### Inclusion and exclusion criteria

All newly diagnosed PMA patients would be consecutively enrolled into our registry platform for ALS and other MND [7, 9] and neuropsychological investigations began in September, 2013. In this present study, PMA patients fulfilled the inclusion criteria proposed by Visser J et al as follows [8]: (1) a disease duration of less than 5 years from the time of symptom onset; (2) clinical and electrophysiological evidence of LMN involvement in two or more of four regions (bulbar, cervical, thoracic and lumbosacral); (3) no conduction blocks on nerve conduction studies; and (4) no clinical UMN signs and symptoms, including forced yawning, crying and laughing, clonus of masseter reflex, (sub)clonic myotatic reflexes, Hoffmann-Trömner sign, extensor plantar response or spasticity. The data of those with history of other neurological conditions which could have an impact on neuropsychological assessments (major stroke, traumatic brain injury, learning disability and severe active epilepsy), alcohol-dependence, drug-dependence, severe active mental illness, use of high-dose psychoactive medication, other mother language instead of Chinese Mandarin and illiteracy were excluded from analysis. 20 ALS patients registered in the platform during the same period and 20 HC were recruited to our hospital, and they were selected in accordance with the same exclusion criteria as PMA patients. Patients with PMA, ALS and HC were matched for age and education level. This study was approved by the Research Ethics Committee of Peking Union Medical College Hospital. Patients visited our clinic accompanied by their legal guardians, and all the patients and HC were included after informed written consent had been obtained from them or their guardians, as set forth by the Declaration of Helsinki.

### Demographic and clinical assessment

Demographic and clinical information including age, gender, level of education, site of symptom onset, disease duration (defined as time lapse between symptom onset and time of neuropsychological evaluation) were collected. Disease severity was assessed by the revised ALS functional rating scale (ALSFRS-R) [10]. To further analyze bulbar function, we then applied swallowing subscale of ALS Severity Scale (ALSSS) [11].

### Neuropsychological assessment

We’ve chosen a series of standardized batteries covering the cognitive domains of executive function, memory, language, attention, visuospatial function and calculation, especially emphasizing the detection of executive and attention dysfunction. Selected tests are as follows: the Mini-Mental State Examination (MMSE) [12]; category and phonemic verbal fluency [13–15]; the Stroop Color-Word Test (SCWT) [16]; the Clock Drawing Test [17] (4 point method was adopted and lower than 4 point was defined as abnormality); paired associate word learning of the Clinical Memory Test (CMT) [18]; episodic memory of modified Wechsler Memory Scale (WMS) [19]; the Symbol Digit Modalities Test [20]; digit span and calculations of the Wechsler Adult Intelligence Scale (WAIS) [21]; copy and repetition subsets from Aphasia Battery of Chinese (ABC) [22]. Stroop interference effect (SIE) was assessed by time (Stroop C time-Stroop B time) and correct number (Stroop B correct number- Stroop C correct number), respectively. The neurobehavioral evaluation was conducted through the interview with caregivers about the patients’ daily performance and the administration of the Frontal Behavioral Inventory-ALS version (FBI-ALS) [23, 24]. It is a 24-item scale originally designed to screen behavioral variant FTLD (bvFTD) and afterwards revised for availability to patients with ALS by adding scripted questions to distinguish behavioral symptoms from physical disability. Depression and anxiety were assessed by Hamilton Depression Rating Scale (HAMD) [25] and Hamilton Anxiety Scale (HAMA) [26].

The batteries were administered within a week after the diagnosis being made, and it required about 2 hours to complete them. So, in order to reduce the impact of fatigue, subjects could choose to take a short break or finish it at the same time next day if necessary, but the tests must be completed in a given sequence to avoid the possible interference of one test over the following ones.

### Case-by-case analysis

By comprehensively analyzing medical history, cognitive and behavioral performance, we obtained a conclusion about whether the patient or the control was demented or not. The Neary criteria for frontotemporal dementia were adopted to diagnose FTLD [27], and the diagnosis of non-FTLD dementia was based on the criteria of the Diagnostic and Statistical Manual of Mental Disorders-IV [28]. To further analyze behavioral change, we adopted he consensus criteria proposed by Strong et al [29] to diagnose ALS/MND with behavioral impairment. We did not make the diagnosis of mild cognitive impairment because several batteries lack normative value for Chinese population.

### Statistical Methods

Continuous values were shown in the form of mean (standard deviation, SD) or median and those categorical ones were in proportion.

Concerning intergroup comparison, χ^2^ test was employed to analyze nominal variables and non-parametric tests were adopted for continuous variables because none of these variables were normally distributed. Specifically, Kruskal-Wallis tests were adopted for the comparison of clinical and demographical characteristics between ALS, PMA and HC. Mann-Whitney tests were used to analyze cognitive performances of PMA vs. ALS, PMA vs. HC and ALS vs. HC, respectively. In this section, we aimed to display in detail intergroup differences of cognitive data, so we chose multiple 2 sample comparison instead of a 3 group test and Mann-Whitney as post hoc. And afterwards Bonferroni correction was made to adjust α value. For the comparison of continuous variables of behavioral and mood status between PMA and ALS, Mann-Whitney tests was also employed.

The level of significance was set at p<0.05. Statistical analyses were carried out using SPSS 11.5 (SPSS Inc).

## Results

**Fig 1:**
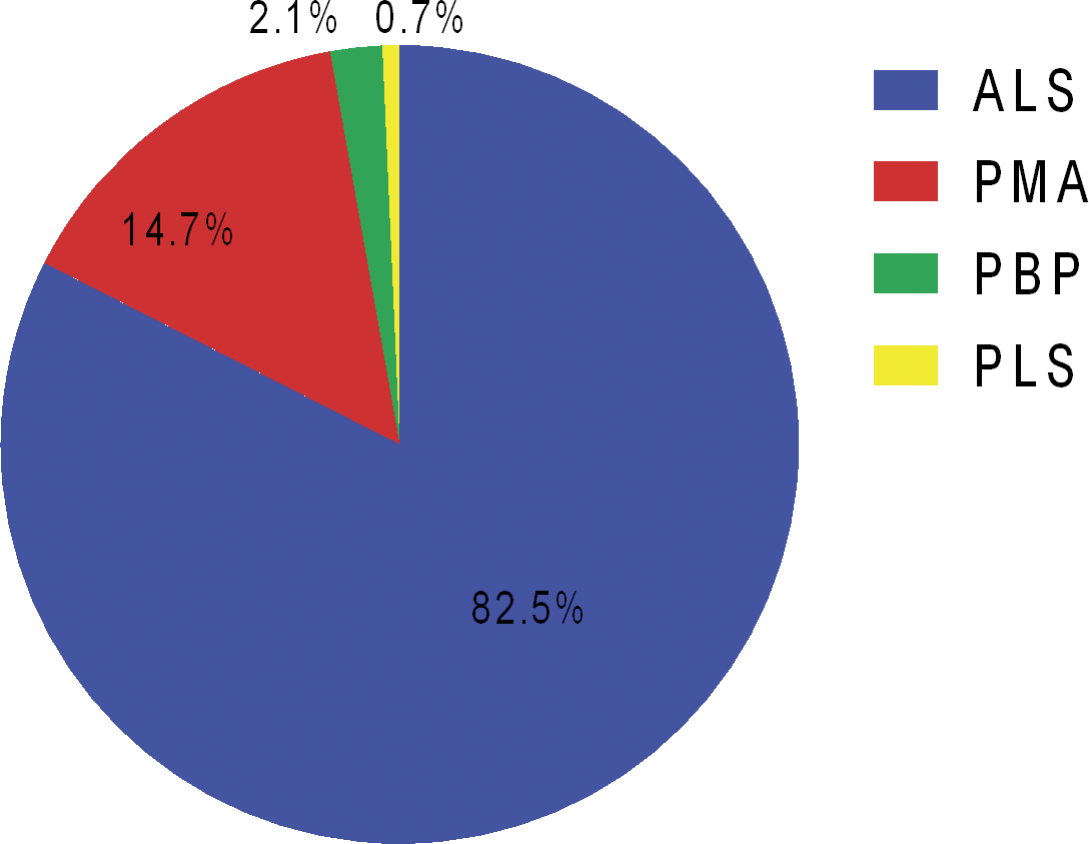
Spectrum of MND registered in Peking Union Medical College Hospital (n = 143). ALS, amyotrophic lateral sclerosis; MND, motor neuron disease; PBP, progressive bulbar palsy; PLS, progressive lateral sclerosis; PMA, progressive muscular atrophy.

From September 1st, 2013 to August 31st, 2014, 21 patients newly diagnosed with PMA were consecutively enrolled into our ALS and MND registry platform, accounting for 14.7% of all the incident MND cases registered during the same period (Fig 1). None of them had a family history of MND, dementia or psychosis. The mean age of PMA patients at the time of diagnosis was 52.6±13.0 years (22.0–74.0 years). 4 patients (19.0%) had bulbar-onset PMA, while the remaining 17 patients (81.0%) had spinal onset disease. Median time from symptom onset to diagnosis was 12 months (range 5–40). At baseline, median ALSFRS-R score was 42 (range 28–47), and median ALSSS swallowing score was 10 (range 7–10). At the time of diagnosis, no one had gastrostomy or non-invasive ventilation. All of them underwent neuropsychological tests, but the data of a 69 year-old female patient was excluded due to illiteracy, thus there were 20 patients in the final analysis. Recruited ALS patients fulfilled the revised El Escorial criteria for clinically definite (2 patients), probable (11 patients), and lab-supported probable ALS (7 cases). Age, education level and gender distribution were not significantly different among PMA, ALS and HC group. Differences of clinical variables, including onset type, function score, disease duration and progression rate were also proved to be not significant between PMA and ALS (Table 1).

**Table 1:** Demographic and clinical characteristics of patients and controls.

	PMA (n = 20)	ALS (n = 20)	HC (n = 20)	p Value
Age at symptom onset (years)	50.6±12.6	50.4±10.5		0.871
Age at evaluation (years)	51.8±12.7	51.6±10.4	51.7±10.7	0.951
Education level (years)	10.4±3.8	10.8±3.7	10.5±3.1	0.876
Gender (male, %)	70	65	45	0.233
Onset type (bulbar, %)	20	15		0.677
Swallowing subscale of ALSSS	9.7±0.9	9.8±0.8		0.655
ALSFRS-R score	40.6±4.8	42.2±3.3		0.313
Disease duration (months)	14.1±9.3	13.8±11.7		0.464
Progression rate	0.6±0.5	0.7±0.5		0.766

ALS, amyotrophic lateral sclerosis; ALSFRS-R, revised ALS functional rating scale; ALSSS, ALS severity scale; HC, healthy controls; PMA, progressive muscular atrophy; the disease progression rate was calculated according to the formula of (48-ALSFRS-R score)/disease duration (month).

Data were means±SD.

In the aspect of cognitive performances, compared to HC, PMA patients performed significantly worse in episodic memory and attention maintenance detected by time of Stroop A and B, whereas ALS patients had significantly longer time for SIE not only than HC but also than PMA. And significant difference also occurred between ALS patients and HC in the MMSE and Stroop B time. However, only difference of Stroop A time between PMA patients and HC, and longer time for SIE of ALS patients than HC and PMA patients survived Bonferroni correction (α = 0.05/3 = 0.017). All three groups did not significantly differ in the cognitive domains of language, visuospatial function and calculation (Table 2).

**Table 2:** Comparison of neuropsychological performances among patients with PMA, ALS and HC.

	PMA	ALS	HC	p Value		
				PMA vs. HC	ALS vs. HC	PMA vs.ALS
MMSE	27.8±1.3	26.7±1.8	28.1±1.1	0.558	**0.020**	0.062
**Executive function**						
Phonemic verbal fluency	6.0±2.6	5.4±1.8	5.1±2.7	0.257	0.303	0.773
Category verbal fluency	15.8±4.1	17.7±4.2	18.1±4.3	0.097	0.946	0.114
Backward digital span of the WAIS	5.1±1.5	4.9±1.6	5.2±1.3	0.725	0.421	0.615
Correct for SIE	1.2±2.3	2.9±3.5	2.1±2.8	0.274	0.520	0.117
Time for SIE	42.7±12.9	62.15±26.4	42.4±15.0	0.819	**0.001** *	**0.003** *
the CDT raw score	3.8±0.6	3.7±0.6	3.8±0.4	0.938	0.655	0.724
Abnormal CDT (%)	20	25	20	1.000	0.705	0.705
**Attention**						
Symbol Digit Modalities Test	41.1±9.9	38.2±11.9	42.3±8.6	0.603	0.255	0.346
Forward digital span of the WAIS	5.1±1.5	4.9±1.6	5.2±1.3	0.832	0.102	0.150
Stroop A time	55.0±13.9	51.3±12.0	44.8±9.1	**0.007** *	0.101	0.626
Stroop B time	81.1±17.9	81.9±21.2	69.8±12.0	**0.049**	**0.035**	0.819
**Memory**						
Paired associate word learning of the CMT	9.4±2.5	9.3±4.6	12.3±3.9	0.125	0.076	0.478
Episodic memory of modified WMS	4.8±1.5	5.4±1.8	6.0±1.5	**0.041**	0.307	0.450
**Language**						
Repetition of ABC	35.5±1.6	35.5±1.7	35.5±1.5	0.496	0.846	0.625
**Visuospatial function**						
Copy of ABC	8.7±2.1	9.2±1.7	9.4±1.0	0.520	0.814	0.764
**Calculation**						
Calculations of the WAIS	13.0±1.6	12.6±3.4	12.3±3.9	0.662	0.849	0.841

3 PMA patients did not complete Stroop Color-Word Task; 2 PMA in copy tests and 1 PMA in digit span and Symbol Digit Modalities Test. ABC, Aphasia Battery Chinese; ALS, amyotrophic lateral sclerosis; CDT, clock drawing test; CMT, Clinical Memory Test; HC, healthy controls; PMA, progressive muscular atrophy; SIE, Stroop interference effect; time for SIE was calculated according to the formula of (Stroop C time-Stroop B time) and correct number for SIE was calculated according to the formula of (Stroop B correct number- Stroop C correct number); WAIS, Wechsler Adult Intelligence Scale; WMS, Wechsler Memory Scale.

Data were means±SD.

*p value remained to be significant after Bonferroni correction (α = 0.05/3 = 0.017).

The FBI-ALS scores were separated into two parts, negative and disinhibitive. ALS patients were not more susceptible to behavioral dysfunction than PMA patients in either subscale, no matter whether it was analyzed quantitatively or proportionately. As shown by HAMA, ALS patients are significantly more prone to anxiety than PMA patients, yet with similar degree in depression shown in HAMD (Table 3). In case-by-case analysis, none of the patients or the HC reached the diagnosis criteria of FTLD or non-FTLD dementia, and one ALS patients and 2 PMA patients could be diagnosed as behavioral impairment.

**Table 3:** Behavioral impairment and mood differences between PMA and ALS.

	PMA (n = 20)	ALS (n = 20)	p Value
**Behavioral impairment**			
FBI-ALS negative	0.8±2.9	1.0±2.4	0.758
FBI-ALS disinhibitive	0.5±1.1	0.1±0.4	0.414
FBI-ALS negative (score≥1, %)	15	20	0.677
FBI-ALS disinhibitive (score≥1, %)	20	5	0.151
**Mood**			
HAMD	2.5±3.1	2.7±3.2	0.495
HAMA	1.9±2.3	4.0±4.1	**0.049**

ALS, amyotrophic lateral sclerosis; FBI, frontal behavioral inventory; HAMA, Hamilton Anxiety Scale; HAMD, Hamilton Depression Rating Scale; PMA, progressive muscular atrophy.

Data were means±SD.

## Discussion

The present study is the first one to describe cognitive and behavioral profile of PMA in a Chinese population. From the perspective of epidemiology, the proportion of PMA in our registry platform represents its actual ranking in MND spectrum [7], although it is a clinic-based design of small sample size. The demographic variables of included patients are similar to those published before [7, 9], and the high response rate has effectively minimized the selection bias. All of these have guaranteed the credibility of this cohort to truly reflect the characteristics of Chinese patients. In the diagnosis link, evidence of 2 LMN regions involved was set as the threshold of inclusion, but indeed, it was shown that all patients were neurophysiologic abnormal in at least 3 regions in electromyography test, which ensured the diagnosis accuracy.

Occupying the largest portion of neurodegenerative MND, ALS was regarded as the key to understanding the relationship between MND and FTLD [30–33]. However, it does not mean that other types of MND are free from cognitive or behavioral impairment. It has been revealed that primary lateral sclerosis (PLS), a purely UMN involved MND with relatively mild disease course, could display the similar pattern of cognitive decline to that of ALS [34], while disputes concerning PMA still remained. Research by Raaphorst J et al indicated that PMA patients showed cognitive deficit especially in executive function and attention/working memory [35], corresponding to their following neuroimaging results of prefrontal cortex activation abnormalities [36], while Wick P et al failed to identify any significant differences between PMA patients and HC on neuropsychological level [37]. In the present investigation, we have also observed several variables implying attention decline in PMA and ALS with only one survival after Bonferroni correction (PMA vs. HC in Stroop A time). Even if the data of patients with dysarthria were excluded from comparison, this difference remained to be significant (p = 0.01). This phenomenon detected by SCWT has seldom been reported in PMA, but in PLS [34], and it possibly indicates that these different phenotypes could be traced to a common origin. The importance of SCWT is not confined to this, although it is not applicable to patients with severe bulbar dysfunction. The only significant difference between PMA and ALS just in the executive area–resistance to interference- was revealed by it, supporting the previous finding of more extensive deficit in ALS [35]. The completion of SCWT required participation of prefrontal cortical areas, especially the dorsolateral prefrontal cortex which was just the vulnerable area of patients with ALS/MND [38, 39]. Also demanding involvement from multiple frontostriatal circuits, verbal fluency has been repeatedly proved to be a sensitive task for executive dysfunction in ALS [30–33]. However, this result was not replicated in the present study.

Intermediate status between cognitive intactness and FTLD also presented as behavioral changes. Compared by mean score of the FBI-ALS, PMA patients did not exhibit a significant milder extent of involvement than ALS in this aspect. Our conclusion might be challenged for its being established on an assumption that HC would nearly score 0 in the FBI-ALS, so we carried out the case-by-case analysis: one ALS could be diagnosed as ALS-behavioral impairment, whereas 2 PMA would receive the same diagnosis if the criteria were applied to them, which was still in favor of the occurrence of frontotemporal syndrome in PMA. Taking into consideration these results and their cognitive performance, none of them could be diagnosed as FTLD or dementia of other types. However, it is inappropriate to regard PMA as a special type resistant to dementia due to equally low rate of comorbid FTLD in ALS of Eastern Asian cohort, probably resulting from C9ORF72 mutation rarely seen in this population [30].

Clinically, ALS and PMA displayed unexpected comparabilities in such variables as onset type, function score, progression rate and so on, considering ALS patients were selected to match PMA ones with age and education in this study. Ruling out the possibility that their neuropsychological comparison was influenced by physical disability, this result might hint the homogeneity of these 2 subtypes. Disease progression rate before diagnosis we presented might be impacted by recall bias, though, it was still of predictive value because of suggested linear progression of ALS [40]. Besides, failing to be proved as an independent protective factor for prognosis, PMA phenotype could have relentless disease course as ALS [8]. Moreover, bulbar-onset patients seemed vulnerable to cognitive impairment and dementia, correlating with relevant cortex hypometabolism in Positron Emission Tomography [33]. We noticed relatively higher prevalence of bulbar-onset PMA in our cohort than previous ones [35, 37], and consequently the similar distribution of this type might serve as an explanation to almost the same neuropsychological performance between ALS and PMA.

Our study has several limitations. Firstly, single center-based investigation would be influenced by possible referral bias to some extent, making it necessary to testify our conclusion in future multicenter-based or population-based design of large sample size. Moreover, neuropsychological tests still waited to be perfected: considering the small proportion of patients with dysarthria, we did not make adjustment to the time-dependent tests which might potentially lower the score of patients with bulbar palsy. The assessment of premorbid IQ was not included in our neuropsychological batteries for avoiding patients’ fatigue. Last but not least, there is a possibility that PMA could turn into ALS with disease progression, and the cross-sectional observation would be inherently incapable of resolving this issue. Future follow-up study and comprehensive analysis of cognitive profile and disease turnover would further substantiate the relationship between ALS and PMA.

In conclusion, we depicted the cognitive and behavioral features of Chinese patients with PMA. They performed significantly worse in Stroop time than HC, providing evidence of attention impairment. When compared to ALS patients, they displayed cognitive dysfunction confined in a relatively smaller area but with similar pattern of behavioral impairment.
